# Transautophagy: Research and Translation of Autophagy Knowledge 2020

**DOI:** 10.1155/2022/9792132

**Published:** 2022-02-21

**Authors:** Nikolai Engedal, Tassula Proikas-Cezanne, Maria C. Albertini, Eva Žerovnik, Jon D. Lane

**Affiliations:** ^1^Oslo University Hospital, Oslo, Norway; ^2^Eberhard Karls University Tübingen, Tübingen, Germany; ^3^International Max Planck Research School “from Molecules to Organisms”, Tübingen, Germany; ^4^University of Urbino Carlo Bo, Urbino, Italy; ^5^Jožef Stefan Institute, Ljubljana, Slovenia; ^6^University of Bristol, Bristol, UK

Autophagy is an evolutionary conserved mechanism for degradation and recycling of cellular material, whose altered activity is associated with aging and a growing list of human pathologies. As a selected annual special issue, the “Transautophagy: Research and Translation of Autophagy Knowledge” series—initiated as part of the objectives of the COST Action Transautophagy (CA15138) aims to collect high-quality papers that disseminate novel findings and new knowledge concerning the molecular mechanisms regulating autophagy and the role of autophagy in human pathologies, in particular those related to oxidative stress and aging.

The current special issue, “Transautophagy: Research and Translation of Autophagy Knowledge 2020”, comprises 8 cutting-edge publications relevant to the above-described themes. Together, the 4 original research papers and 4 review papers convey important new insights into (i) novel mechanisms of signal control of autophagy by hypoxia, ataxia telangiectasia mutated protein kinase (ATM), the ER stress sensor inositol-requiring enzyme 1 (IRE1), and the process of O-GlcNAcylation; (ii) the role of autophagy in various age-related diseases such as cardiac fibrosis, heart failure, Parkinson's disease, intervertebral disc degeneration (IVDD), and age-related macular degeneration (AMD); and (iii) the role of autophagy in cancer, with a particular focus on resistance mechanisms to anticancer therapy and biomarkers of chaperone-mediated autophagy (Figure 1).

ATM is best known as a mediator of DNA damage-induced signaling. However, recent evidence point towards a more versatile role of ATM in sensing different kinds of stress signaling, including that arising from oxidative stress. M. Blignaut et al. discuss the crosstalk between oxidative stress as an activator of ATM and its potential role as a master regulator of general autophagy as well as of various types of selective autophagy (mitophagy, pexophagy, and aggrephagy). The review includes a special focus on terminally differentiated cells like neurons and cardiomyocytes. In terms of the latter, autophagy has been implicated in cardioprotective responses. J. Qu et al. examined the role of Sigma-1 receptor (Sig1R), a chaperone in the endoplasmic reticulum (ER) membrane, in cardiac fibroblast activation in a mouse model, as well as in neonatal rat cardiac fibroblasts. Their results indicate that Sig1R plays a protective role in the activation of cardiac fibroblasts by inhibiting the IRE1 pathway and restoring autophagic flux. Sig1R may therefore represent a therapeutic target for cardiac fibrosis. The cellular effects of O-GlcNAcylation have received increased attention over the recent years. H. Yu et al. employed an O-linked *β*-N-acetylglucosamine transferase (OGT) cardiomyocyte-specific knockout mouse model for the first time. They provided data to indicate that O-GlcNAcylation is required for autophagy in cardiomyocytes and that O-GlcNAcylation of the central autophagy-initiating kinase ULK1 may stimulate its activity towards promoting autophagy. Together, these three papers provide novel insight into signal control of autophagy and molecular mechanisms underlying the association between stress responses, autophagy, and heart disease.

Subsequent papers in this special issue have explored the relationship between autophagy and other age-related conditions. N. Jimenez-Moreno and J. D. Lane reviewed the molecular machinery of the autophagic pathway as a background to analyse the crosstalk between autophagy and redox homeostasis and their role in neurodegenerative diseases, with a particular emphasis on the pathogenesis of Parkinson's disease. They describe how dysfunctional autophagy typically correlates with neurodegenerative diseases, and that mitochondrial dysfunction, with associated increases in oxidative stress, and declining proteostasis control are likely to be key contributors to Parkinson's. Intervertebral disc degeneration (IVDD) is a common cause of lower back pain. H.-J. Kim et al. hypothesized that the nucleus pulposus cells that make up the center of the intervertebral disc can be affected by aging and environmental oxygen concentration, thus affecting the development of IVDD. Their results from analyses of isolated nucleus pulposus cells from rat lumbar discs suggest that nucleus pulposus cells modulate the expression of chondrogenesis-, autophagy-, and apoptosis-related genes under hypoxic conditions. The study also provided hints to changes that occur in nucleus pulposus cells during aging. Age-related macular degeneration (AMD) is a major cause of visual loss and irreversible blindness in the elderly population worldwide. Z.-Y. Zhang et al. describe how age-related, cumulative oxidative stress contributes to the pathogenesis of AMD. Furthermore, they discuss how autophagy can prevent oxidative damage in AMD by protecting retinal pigmental epithelial cells and photoreceptor cells from degeneration and death. Finally, they review potential neuroprotective strategies for therapeutic interventions and provide an overview of such neuroprotective mechanisms.

Cancer is another pathology that is frequently associated with aging and oxidative stress. A. K. Verma et al. provide an overview of the autophagic pathway and its regulation as a framework to discuss the dual role of autophagy in cancer, followed by a focused review of the role of autophagy in resistance mechanisms to anticancer therapy and strategies to modulate autophagy in order to overcome resistance. Different types of autophagy exist, and while the process of autophagosome-associated autophagy (macroautophagy and autophagosome-associated selective autophagy) has been most extensively studied, much less is known about the chaperone-mediated autophagy (CMA) pathway. CMA is characterized by the direct import of cytosolic proteins into degradative lysosomes, in a process where LAMP2A and HSC70 are essential players. T. Losmanová et al. examined the expression LAMP2A and HSC70 in pulmonary squamous cell carcinoma (pSQCC) tissue from 336 patients and compared the expression to clinical data. They reported that high LAMP2A or HSC70 expression was associated with worse outcome, including overall survival. The authors suggested that elevated levels of LAMP2A or HSC70 could be indicative of increased CMA activity and that these two proteins might be useful potential biomarkers for future CMA-inhibiting therapies.

In summary, this annual special issue has disseminated important novel developments in the field of autophagy and oxidative stress research and provides more understanding of molecular and cellular mechanisms of different diseases with emphasis on translation of knowledge to treatment, including topics of autophagy regulation and oxidative stress in aging, age-related diseases, neurodegenerative diseases, and cancer.

## Figures and Tables

**Figure 1:**
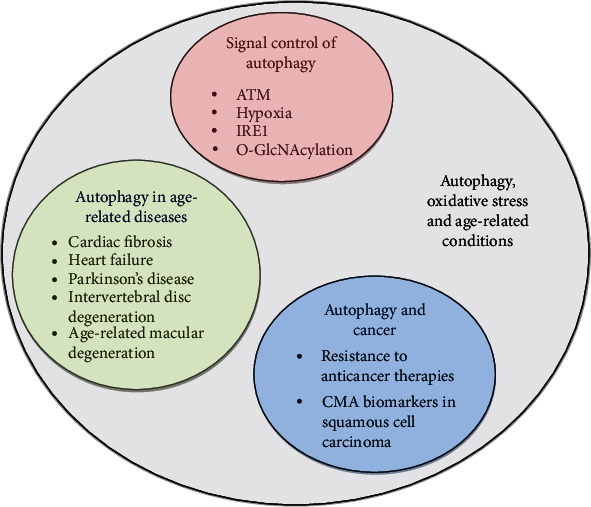
Summary of topics that have been discussed in review articles or have been addressed in original studies in this special issue. Abbreviations: ATM: ataxia telangiectasia mutated protein kinase; IRE1: inositol-requiring enzyme 1; CMA: chaperone-mediated autophagy.

